# Sleep in the first 1,800 days of life: a critical factor in health and development that deserves more attention

**DOI:** 10.3389/frsle.2024.1440677

**Published:** 2024-09-04

**Authors:** Stephen H. Sheldon, Judith Owens

**Affiliations:** ^1^Emeritus, Pediatrics and Neurology, Northwestern University Feinberg School of Medicine, Emeritus, Pediatric Sleep Medicine Center, Ann & Robert H. Lurie Children's Hospital of Chicago, Chicago, IL, United States; ^2^Department of Neurology, Harvard Medical School, Boston Children's Hospital, Boston, MA, United States

**Keywords:** pediatrics, sleep, neurodevelopment, synaptogenesis, brain maturation

## Introduction

Over the past 25 years, pediatric and adolescent sleep and circadian medicine has paralleled evolution of health care for infants, children, and adolescents in the United States (Sheldon, 2024). Sleep medicine has become an imperative and major medical discipline for adults and has provided insight into the future direction for clinical practice and research in pediatric sleep medicine. The following opinion piece, while focused primarily on data from the US, is intended to make a cogent argument for elevating the importance and status of sleep medicine in children and adolescents globally to equal that of our adult counterparts. We also acknowledge the substantive worldwide contribution to pediatric sleep medicine education and research already made by numerous individuals and by international organizations such as the World Sleep Society (WSS), the European Sleep Research Society (ESRS0 and the International Pediatric Sleep Association (IPSA).

Most animals sleep more early in life when compared to total sleep time in adulthood. Humans seem to have a longer developmental childhood than most other mammals. Sleep has also been depicted in art and literature for millennia. However, the science of sleep is not in its infancy but in its neonatal period, especially with regard to children. While adult sleep medicine has only been recognized as a legitimate clinical and research specialty for < 50 years, pediatric sleep medicine has lagged further behind; for example the first published description of obstructive sleep apnea in children was in 1975 by Dr. Christian Guilleminault and the first pediatric sleep clinic in the US was established by Dr. Richard Ferber around the same time.

Although “children are not just short adults” is cliché, the clinical focus has traditionally been on standards, procedures, and diagnostic criteria originally created for the adult patient, then seeing how these criteria might be adapted for children. As a result, the lack of standardization of methods for conducting pediatric sleep diagnostic services and providing comprehensive evaluation and treatment of pediatric sleep disorders, including in “mixed” adult and pediatric centers, greatly increases the risk of delivery of “second class” or substandard care for children (Owens et al., 2012). An American Academy of Sleep Medicine (AASM) online survey of revealed that 28 named pediatric sleep centers accounting for only 1.8% of the accredited sleep centers in 2012 (Sheldon, 2012). Of all centers reviewed, only 47% accepted children 5 years of age or younger. Less than one-third evaluated and managed children < 3 years of age. Census data from 2020 revealed ~22 million children < 5 years of age. Although there has been a significant increase in accredited sleep centers, proportions remain about the same. There is clearly a significant service gap and poor access to care for specialized pediatric sleep medicine services in both academic and non-academic settings. Sites for neonatal and pediatric sleep and circadian research are considerably lesser in number. This service gap should also be viewed in the context of the substantial prevalence of a variety of sleep disorders in the pediatric population (e.g., insomnia in 10–30% of children overall (American Academy of Sleep Medicine, 2023); obstructive sleep apnea in 1–4% (Bitners and Arens, 2020); and delayed sleep-wake phase disorder in up to 8% of adolescents (Duffy et al., 2021).

In 1968, Rechtschaffen and Kales (1968) published the seminal guide for evaluating sleep in the adult patient. At the time, the development of a similar guide for evaluating sleep in children was deemed too difficult and deferred. Despite the challenges, in 1972 Anders et al. (1971) published a similar manual for standardization of evaluating sleep in neonates. However, a focus on sleep in infants beyond the neonatal period, and in toddlers, and young children was generally not considered as a priority the AASM, American Academy of Pediatrics (AAP), or the American Board of Pediatrics (ABP); failing perhaps at the time to recognize the basic fact that in this age group, *individuals normally spend more hours asleep than awake, testifying to the critical importance of sleep in neurodevelopment, physical growth and general health*.

In order to address this historical lack of appropriate attention to the science and practice of sleep in children, in 2023, a White Paper was published by the Sleep Research Society (SRS) addressing current knowledge, gaps, and opportunities for the future of Pediatric Sleep research and clinical practice (Reynolds et al., 2023). Current knowledge, scope, and direction for basic and clinical research was presented. Epidemiology, development of sleep and circadian rhythms, as well as sleep disorders were reviewed, and a practical roadmap for future research was presented. Nonetheless, there was only brief mention of neurodevelopmental aspects and the importance of sleep (and/or its bidirectional influence). Little was discussed of the importance of neural development, neural network formation and maintenance, synaptic growth, dendritic branching, synapse stability/strength, neural plasticity, and synaptic pruning. Therefore, while this White Paper did an admirable job of summarizing the current state of understanding and practice of pediatric sleep medicine, the significance of normal sleep on development of the central nervous system still received less attention than deserved.

## Sleep's critical role in early neurodevelopment

**Figure 1:**
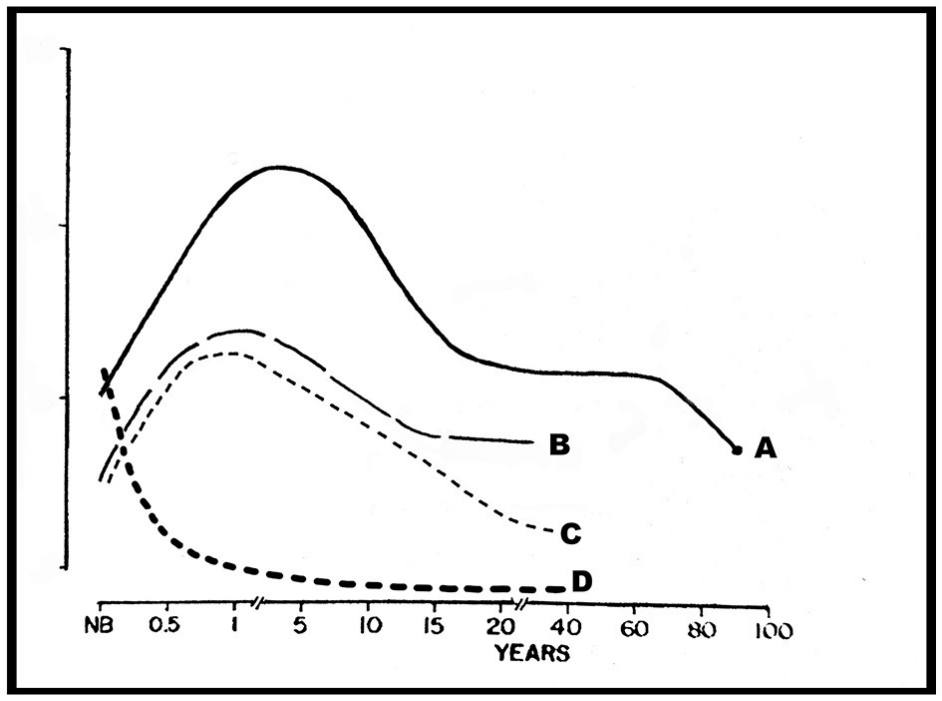
Representation of neuronal and synaptic densities in layer 3 of the frontal cortex and slow wave sleep density across the lifespan. A, synapses/mm^3^ × 10^8^; B, slow wave sleep density; C, synapses/neuron × 10^4^; D, total neuron density/mm^3^ × 10^4^. Modified from Huttenlocher (1979) and Kurth et al. (2015).

In the 1970's Huttenlocher (1979) studied the development of synapses and synaptic density in the frontal cortex across the lifespan. Notably, synaptic development at birth was quite high and almost as dense as in the adult brain (~12.5 × 10^8^ per mm^3^). Surprisingly, from 6 months to 7 years of age synaptic density increased to its maximum (~14.8 × 10^8^ per mm^3^). Subsequently, synaptic density decreased to the adult density and remained at that level from 16 to 72 years of life. Immature synaptic profiles were morphologically distinguishable from mature profiles. There were continuous presynaptic bands of irregular width in the immature profile compared to well-defined polygonal shapes of the mature synapses indicating synaptic maturation. Nonetheless, synaptic density reached its maximum during the first 1,800 days of life and decreased thereafter, remaining stable throughout the remainder of the lifespan. Other sleep-related physiological functions showed similar peaks occurring during the first 5 years of life (see Figure 1).

Although all animals sleep, the exact function of this ubiquitous state remains unknown. Kurth et al. (2015) reviewed multiple issues regarding sleep and cortical development. Ontogenesis of sleep state, development of sleep architecture, sleep regulation, cortical development, white matter development, and the development of CNS energy used during development were reviewed, and hypotheses and remaining research questions were presented. It was hypothesized that sleep is important for balancing synaptic strength, shape, and brain plasticity. Neuronal density is high in the neonatal brain and falls rapidly during the first 6 months of life (Huttenlocher, 1979). Between 1 and 2 years of life density remains unchanged. However, density decrement was smaller and gradual with neuronal density about 55% greater than the mean synaptic density at 2–3 years of age and 10% above the mean synaptic density at 7 years of age.

There seems to be sleep-dependent improvement in brain function and performance. Sleep is also needed for plasticity associated with neurodevelopment (Kayser et al., 2014), changes in synapse size and morphology, and malleability of existing synapse connections. Finally, sleep increases interstitial space apparently providing a mechanism for reducing neurotoxic waste (Xie et al., 2013).

Simola and colleagues, in a 4-year follow up study, evaluated psychosocial and somatic outcomes of sleep problems in children. Insufficient sleep relates to medical and psychosocial problems. Persistent sleep problems in children related to a 16-fold increased risk of having psychosocial symptoms compared to children without sleep problems. Somatic complaints and medical problems had a 9-fold greater risk (Simola et al., 2014).

## A call to action

Based on these data, both scientific and observational, the importance of attaining a more comprehensive understanding of sleep in infants and young children during the first 1,800 days of life is clear. Presently, the importance of sleep in a developing human who is normally asleep more than awake lends intuitive credence to the prominence of sleep as it relates to neurological development in particular. In addition, a paradigm shift in focus in pediatric medical science is of universal importance for both children and adults, as it may well-shed light on the development of neurological deficits and degenerative diseases across the lifespan. Even minor disruption to this intricate system might result in long term disability, albeit not directly visible, but nonetheless dysfunctional. This, data regarding early development in synaptic density, neuronal density, and many other biological and neurophysiological variables that peak during the first 1,800 days of life potentially has enormous implications for the identification and management of sleep-related disorders and disrupted sleep. Furthermore, such factors as the consequences of intermittent oxygen deprivation during sleep, carbon dioxide accumulation during sleep, inflammatory consequences of sleep fragmentation, sleep state disruption, state progression, and disruption/dysfunction of circadian development during this period of neural development may result in consequences that may not be reversible. Kocevska et al. (2017) demonstrated children with more sleep disturbances in the first 6 years of life had smaller gray matter volumes at age 7 years. Yet the current answers to these critical questions are often not evidence-based and much of the hypotheses are based in speculation.

Public health implications are also significant. It has been shown that disrupted sleep and insufficient sleep can result in medical and psychosocial consequences. These may be addressed and subsequently resolved in older children and adults. But, if present and unattended to before 7 years of age, complexity, distribution, neural network development, synaptic strength, and normal pruning may be impacted significantly enough to affect quality of life of the child for many years, or perhaps permanently.

There is, therefore, a dire need for increasing the number of child centered sleep researchers and clinicians in order to reach a critical mass of child health care professionals who address knowledge gaps in sleep and circadian sciences. We need to engage trainees across multiple disciplines, including neurology and neuroscience, and at various training levels to consider a research focus on sleep in the first 1,800 days of life. Funding from government and private foundation sources for expansion of the pool of talented young researchers interested in exploring the impact of sleep and circadian rhythms on early development is critical. While the need is great, the rewards reaped from these investments in time, money and mentorship have enormous potential for positive change.

Examination for certification in Sleep Medicine was first administered in the US in 2007 and was taken by 1,882 candidates, of which 78 (4%) were pediatricians (American Board of Pediatrics candidates) (Quan et al., 2008). This certification examination was administered every 2 years. In 2011 a total of 2,457 candidates were admitted (Quan et al., 2012). Although there was an increase of 31% in those taking the Sleep Medicine certification examination, only 71 (3%) were certified by the America Board of Pediatrics (ABP). A total of 3% were admitted by the American Academy of Family Medicine and 1% by the American Board of Anesthesiology (ABA). In contrast, American Board of Internal Medicine examined 1,512 (62%), American Board of Psychiatry and Neurology 527 (21%), and the American Board of Otolaryngology 108 (4%) admitted more candidates.

According to 2020 data, there are 679.15 million children in the world < 5 years of age. According to the 2020 US Census, there are 22.4 million children between 0 and 5 years of age and another 24.2 million children between the ages of 6 and 11 years. With only 479 sleep medicine certified pediatricians. *In the US, this translates to 1 certified pediatric sleep specialist for every 46,764 children under 5 years of age*!

In 2017, there were 5,800 board certified sleep specialists in the US. Of these, only 270 were pediatricians or pediatric sub-specialists (Kravitz, 2017). Of these pediatricians, 51% were general pediatricians and only 2 (0.4%) were also certified in neonatal-perinatal medicine and 2 (0.4%) were also certified in developmental and behavioral pediatrics. Clearly, there is a dire need to recruit and train young researchers and clinicians to this foundational and exhilarating field of exploration and service.

## Conclusions

Given the unique characteristics, prevalence and potential long-term consequences of deficient sleep in young children on brain development, performance, health and safety well into adulthood, it is imperative that we recognize and address the substantial gaps in clinical services, education and research in pediatric sleep that exist worldwide, especially in regions of the world that lack the resources to address these gaps. Developing and fostering education and training in pediatric sleep medicine for health providers requires substantive commitment, funding and mentorship for less developed countries through initiatives such as the WSS World Sleep Academy. Pediatric Sleep professional organizations such as IPSA have begun to develop efforts to “jumpstart” clinical research by offering startup grants to newer investigators in resource-poor regions. In particular, we need additional prospective longitudinal data to further assess the linkage between sleep in the first 1,800 days and poor outcomes, as well as to identify modifying factors that may increase relative vulnerability or resistance to these effects in a variety of populations. Ultimately, it is our responsibility as sleep health advocates for children and families to promote the field of pediatric sleep medicine for the benefit of all.
